# Evaluation of Trends in Oncology Drug Spending in Medicare, 2016 to 2020

**DOI:** 10.1001/jamanetworkopen.2022.21468

**Published:** 2022-07-13

**Authors:** Michael Anne Kyle, Stacie B. Dusetzina, Nancy L. Keating

**Affiliations:** 1 Department of Health Care Policy, Harvard Medical School, Boston, Massachusetts; 2 Department of Health Policy, Vanderbilt University Medical Center, Nashville, Tennessee; 3 Vanderbilt-Ingram Cancer Center, Nashville, Tennessee; 4 Division of General Internal Medicine, Brigham and Women’s Hospital, Boston, Massachusetts

## Abstract

This cross-sectional study examines trends in drug spending in Medicare Parts B and D and describes the share of total use and spending attributed to oncology drugs within each program.

## Introduction

Oncology prescription drug spending concerns policy makers, physicians, and patients. Historically, anticancer medications were delivered via infusion and were covered under a patient’s medical benefit (Medicare Part B). Recent decades have seen the rise of highly effective, orally administered anticancer drugs for certain cancer types covered under outpatient prescription drug benefits (Medicare Part D), for which very high out-of-pocket costs may contribute to financial toxicity.^1,2^ We examined drug spending in Medicare Parts B and D to document trends in oncology drug entrants, use, and spending over time and described the share of total use and spending attributed to oncology drugs within each program.

## Methods

This cross-sectional study incorporated data from the 2016-2020 Medicare Parts B and D Drug Spending Dashboard Public Use Files (eMethods 1 in the Supplement). We used the Oncology Care Model drug lists,^3^ current through 2021, to identify oncology drugs. Because the study did not involve human participants, approval was waived by the Harvard Medical School Institutional Review Board. This study followed the STROBE reporting guideline.

For each program and year, we calculated the following measures overall and for all drugs, oncology drugs, and nononcology drugs: number of drugs, total spending, total claims, total unique beneficiaries using each drug (the metric reported in the dashboards), and mean and median spending per claim and beneficiary-drug combination. We adjusted Part B data using Medicare enrollment percentages to account for missing Medicare Advantage beneficiaries, whose utilization patterns and cancer prevalence are consistent with those of traditional Medicare beneficiaries (eMethods 2 and eTable 1 in the Supplement).^4,5^ Using the total number of oncology drugs in Parts B and D as the denominator, we calculated the share of drugs, claims, and spending across each program (eMethods 3 and eTable 2 in the Supplement).

## Results

Between January 1, 2016, and December 31, 2020, the share of all Part B drugs used for oncology indications increased slightly from 97 of 484 (20.0%) to 136 of 603 (22.5%) (Table). Among beneficiaries receiving Part B drugs, the proportion receiving oncology drugs was unchanged (from 3.0% to 3.2%). During the same period, the oncology proportion of Part B drug spending increased from 33.7% to 43.1%.

**Table.** Allocation of Oncology Drugs, Utilization, and Spending in Medicare Parts B and D, 2016 to 2020^a^

Variable	Study year				
	2016	2017	2018	2019	2020
Oncology share of Part B^b^					
Total No. of Part B drugs	484	497	516	568	603
Oncology share, No. (%)	97 (20.0)	104 (20.9)	108 (20.9)	122 (21.5)	136 (22.5)
Total No. of Part B drug claims	92 708 040	97 252 065	101 913 961	106 157 598	101 778 473
Oncology share, No. (%)	7 485 176 (8.1)	7 804 236 (8.0)	8 172 177 (8.0)	9 277 128 (8.7)	9 257 668 (9.1)
Total No. of Part B beneficiaries^c^	50 922 721	54 805 368	58 624 416	60 504 465	58 621 161
Oncology, No. (%)	1 544 821 (3.0)	1 592 961 (2.9)	1 637 081 (2.8)	1 925 964 (3.2)	1 902 480 (3.2)
Total Part B spending, US$	38 411 070 407	43 122 299 994	48 748 945 517	59 419 202 832	64 094 270 714
Oncology share, US$ (%)	12 929 256 718 (33.7)	15 687 790 907 (36.4)	18 384 773 939 (37.7)	25 224 491 427 (42.5)	27 652.141 787 (43.1)
Oncology share of Part D^d^					
Total No. of Part D drugs	2721	2910	3113	3343	3576
Oncology share, No. (%)	84 (3.1)	96 (3.3)	113 (3.6)	128 (3.8)	141 (3.9)
Total No. of Part D claims	1 397 610 492	1 446 158 469	1 483 268 968	1 501 999 811	1 497 537 926
Oncology share, No. (%)	8 482 508 (0.6)	8 968 233 (0.6)	9 464 530 (0.6)	9 759 482 (0.6)	9 914 511 (0.7)
Total No. of Part D beneficiaries^c^	378 718 110	391 857 035	405 052 770	420 502 427	409 264 437
Oncology share, No. (%)	2 107 236 (0.5)	2 203 496 (0.6)	2 309 205 (0.6)	2 415 515 (0.6)	2 423 464 (0.6)
Total Part D spending, US$	141 224 113 084	151 959 718 705	167 065 058 170	183 032 542 314	198 650 307 576
Oncology share, US$ (%)	12 801 913 363 (9.1)	15 251 300 794 (10.0)	18 875 770 540 (11.3)	22 279 551 395 (12.2)	26 198 193 172 (13.2)
Oncology combined Parts B and D					
Total No. of unique B plus D oncology drugs	181	200	221	250	277
Part B share, No. (%)	97 (53.6)	104 (52.0)	108 (48.9)	122 (48.8)	136 (49.1)
Part D share, No. (%)	84 (46.4)	96 (48.0)	113 (51.1)	128 (51.2)	141 (50.9)
Total No. of B plus D oncology drug claims	15 967 684	16 772 469	17 636 707	19 036 610	19 172 179
Part B share, No. (%)	7 485 176 (46.9)	7 804 236 (46.5)	8 172 177 (46.3)	9 277 128 (48.7)	9 257 668 (48.3)
Part D share, No. (%)	8 482 508 (53.1)	8 968 233 (53.5)	9 464 530 (53.7)	9 759 482 (51.3)	9 914 511 (51.7)
Total No. of B plus D oncology beneficiaries^c^	3 652 057	3 796 457	3 946 286	4 341 479	4 325 944
Part B share, No. (%)	1 544 821 (42.3)	1 592 961 (42.0)	1 637 081 (41.5)	1 925 964 (44.4)	1 902 480 (44.0)
Part D share, No. (%)	2 107 236 (57.7)	2 203 496 (58.0)	2 309 205 (58.5)	2 415 515 (55.6)	2 423 464 (56.0)
Total B plus D oncology drug spending, US$	25 731 170 081	30 939 091 701	37 260 544 479	47 504 042 822	53 850 334 959
Part B share, US$ (%)	12 929 256 718 (50.2)	15 687 790 907 (50.7)	18 384 773 939 (49.3)	25 224 491 427 (53.1)	27 652 141 787 (51.3)
Part D share, US$ (%)	12 801 913 363 (49.7)	15 251 300 794 (49.3)	18 875 770 540 (50.7)	22 279 551 395 (46.9)	26 198 193 172 (48.7)

^a^ Oncology drugs are defined based on the Oncology Care Model list (with National Drug Codes and Healthcare Common Procedure Coding System codes current through 2021). Drugs are mutually exclusively attributed to Part B or D.

^b^ Part B beneficiaries and spending totals were adjusted to account for percentage of Medicare Advantage enrollment in that year (eMethods 2 in the Supplement).

^c^ Total drug beneficiaries is the number of individual beneficiaries filling a drug at least once during the plan year, summed over all drugs (metric provided in Dashboard).

^d^ Part D includes data for all Medicare beneficiaries who choose to enroll (standalone prescription drug plans and Medicare Advantage), which is approximately 77% of total Medicare beneficiaries.

The share of all Part D drugs used for oncology indications increased slightly from 84 of 2721 (3.1%) to 141 of 3576 (3.9%) from 2016 to 2020 (Table). Although the proportion of Part D beneficiaries receiving oncology drugs remained consistent at 0.6%, the proportion of Part D drug spending on oncology drugs increased from 9.1% to 13.2% from 2016 to 2020.

At the beneficiary-drug level, Part B spending increased modestly overall and for nononcology drugs, but median annual oncology drug spending per beneficiary increased from $9325 (IQR, $750-$29 256) to $18 761 (IQR, $1197-$39 335) from 2016 to 2020 (Figure). Part D median per beneficiary oncology drug spending accelerated similarly from $27 761 (IQR, $2088-$53 834) to $52 016 (IQR, $6472-76,894) from 2016 to 2020.

**Figure.**
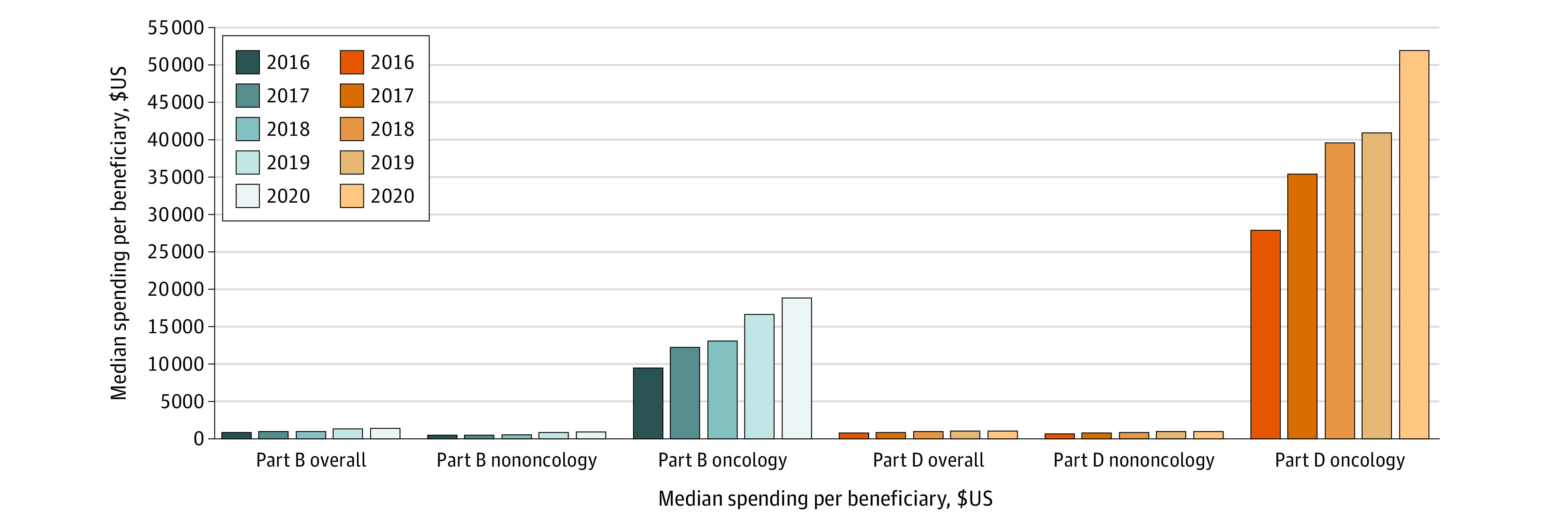
Median Drug Spending per Beneficiary 2016-2020, by Medicare Program and Type of Drug Mean spending per beneficiary is calculated for each drug, dividing the total spending for that drug by total number of unique beneficiaries using that drug.

The proportion of oncology drugs covered by Parts D vs B increased slightly over time. Of 181 oncology drugs in 2016, 84 (46.4%) were Part D. Of 277 drugs in 2020, 141 (50.9%) were Part D (Table).

## Discussion

Within Medicare Parts B and D, the oncology percentage of total drugs and percentage of beneficiaries using oncology drugs were relatively stable, but oncology drug spending increased markedly between 2016 and 2020, both overall and per beneficiary. Study limitations include an inability to disaggregate off-label or nononcology uses of drugs. Nevertheless, these findings underscore the need for attention to the accelerating costs of oncology drugs—particularly oral drugs—and diminishing affordability for patients and the Medicare program.

For Medicare beneficiaries with cancer, Part D accounts for an increasing share of new drugs whose high and rising out-of-pocket costs may contribute to financial toxicity and noninitiation of or nonadherence to oral therapies.^6^ This has consequential policy implications: efforts to cap patient out-of-pocket spending in Part D may disproportionately benefit people with cancer, given these coverage dynamics.

## Notes

Supplement. **eMethods 1.** CMS Medicare Drug Spending Public Use Files (PUF) Variables **eMethods 2.** Adjusting for Medicare Advantage Enrollment **eTable 1.** Medicare Advantage Share of Total Medicare Program Enrollment **eMethods 3.** Count of Oncology Drugs and Mutually Exclusive Assignment to Parts B and D **eTable 2.** Primary Program Assignment for Overlapping Drugs
eReferences
