# Supplementary material for: Evaluation of Trends in Oncology Drug Spending in Medicare, 2016 to 2020
Source: JAMA Netw Open. 2022 Jul 13;5(7):e2221468. doi: 10.1001/jamanetworkopen.2022.21468 (PMC9280395; doi:10.1001/jamanetworkopen.2022.21468)
Supplement: Supplement. — eMethods 1. CMS Medicare Drug Spending Public Use Files (PUF) Variables eMethods 2. Adjusting for Medicare Advantage Enrollment eTable 1. Medicare Advantage Share of Total Medicare Program Enrollment eMethods 3. Count of Oncology Drugs and Mutually Exclusive Assignment to Parts B and D eTable 2. Primary Program Assignment for Overlapping Drugs eReferences [file jamanetwopen-e2221468-s001.pdf]

Supplementary Online Content

Kyle MA, Dusetzina SB, Keating NL. Evaluation of trends in oncology drug spending in Medicare, 2016 to 2020. *JAMA Netw Open*. 2022;5(7):e2221468. doi:10.1001/jamanetworkopen.2022.21468

eMethods 1. CMS Medicare Drug Spending Public Use Files (PUF) Variables
eMethods 2. Adjusting for Medicare Advantage Enrollment
eTable 1. Medicare Advantage Share of Total Medicare Program Enrollment
eMethods 3. Count of Oncology Drugs and Mutually Exclusive Assignment to Parts B and D
eTable 2. Primary Program Assignment for Overlapping Drugs
eReferences

This supplementary material has been provided by the authors to give readers additional information about their work.

© 2022 Kyle MA et al. *JAMA Network Open*.

eMethods 1. CMS Medicare Drug Spending Public Use Files (PUF) Variables

This analysis uses the Centers for Medicare and Medicaid Services (CMS) Drug Spending Public Use Files (PUF) for Parts B and D. These files are reported at the individual drug level, and report: total spending on each drug, total number of claims for each drug, total number of beneficiaries using each drug, average spending per claim, and average spending per beneficiary. The Part Drug Spending PUF includes all Medicare Part D beneficiaries in the given year and is based on the Part D Prescription Drug Event (PDE) file. The Part B Drug Spending PUF includes all drugs Healthcare Common Procedure Coding System (HCPCS) paid in Medicare claims; see Appendix B for more detail.

Total drug spending reports gross drug costs inclusive of spending by the Medicare program and beneficiary liability; it does not include manufacturer rebates. Evidence suggests that oncology drugs in Medicare have small or no rebates, averaging 2% of expenditures according to a recent report of the Government Accountability Office.[1]

Total claims sums the total number of prescription fills for that drug in the beneficiary year.

Total beneficiaries sums the total number of beneficiaries filling a prescription for that drug in the beneficiary year. Note that this means that beneficiaries are counted for each drug they fill. The total number of beneficiaries is thus not a unique count of beneficiaries but the count of unique beneficiary-drug combinations.

Average spending per claim reports the mean spending per claim (prescription fill) for a drug; in this analysis we focus on median spending given the skewed distribution.

Average spending per beneficiary reports the mean per-beneficiary spending on a drug; in this analysis we focus on median spending given the skewed distribution.

© 2022 Kyle MA et al. *JAMA Network Open*.

eMethods 2. Adjusting for Medicare Advantage Enrollment

The Part D Dashboard includes all enrollees in that program, however the Part B Dashboard excludes Medicare Advantage beneficiaries. This is a limitation of the dataset. Evidence suggests that the Medicare Advantage and traditional Medicare populations are similar, including specifically in oncology (37% of beneficiaries in both groups).[2] Recent evidence shows some instances of differences in oncology prescribing patterns in traditional Medicare vs. Medicare Advantage[3], however this study examined 4 non-oncology treatments for which a clear lower-cost alternative exists. Such situations are infrequent for Part B oncology drugs. Futher, a preponderance of program-level data indicate that traditional Medicare and Medicare Advantage do not have statistically significant differences in physician-administered drug spending.[4]

To account for Medicare Advantage members in Part B total spending, total claims, and total beneficiaries, we inflated the Part B beneficiary enrollment numbers and total spending to account for the Medicare Advantage population. We used CMS' report of the Medicare Advantage share of total Medicare enrollment for each year.

eTable 1. Medicare Advantage Share of Total Medicare Program Enrollment

**Year**	**2016**	**2017**	**2018**	**2019**	**2020**
MA % of total Medicare enrollment	32.24	33.85	35.55	37.29	39.89%

Source: https://data.cms.gov/summary-statistics-on-beneficiary-enrollment/medicare-and-medicaid-reports/medicare-total-enrollment

© 2022 Kyle MA et al. *JAMA Network Open*.

eMethods 3. Count of Oncology Drugs and Mutually Exclusive Assignment to Parts B and D

Each dashboard reports unique drugs billed to Part B or Part D, respectively. We used the CMS Oncology Care Model (OCM) triggering drug list to identify drugs as "oncology" based on National Drug Codes (NDC) and Healthcare Common Procedure Coding System (HCPCS).[5] This list is updated to account for new entrants and changes in HCPCS codes; the list we used to identify oncology drugs includes HCPCS codes current through 2021, accounting for all years in this analysis. We conducted sensitivity analyses assessing the impact of top-selling oncology drugs with non-oncology indications (bevacizumab and rituximab). These analyses did not meaningfully change our findings.

Some drugs are billed to both Parts B and D. To avoid double counting the number of drugs, we assigned drugs mutually exclusively to each program based on the preponderance of utilization and Medicare coverage rules for oral anticancer drugs.[1] Most instances were Part B drugs billed in Part D, which was a low-volume occurrence. The list below indicates the program assignment for drugs appearing in both Part B and D spending.

eTable 2. Primary Program Assignment for Overlapping Drugs

**Drug name**
**Part B primary**
Injection, ado-trastuzumab emtansine, 1 mg
Injection, alemtuzumab, 1 mg
Aminolevulinic acid hcl for topical administration, 20%, single unit dosage form (354 mg)
Aminolevulinic acid hcl for topical administration, 10% gel, 10 mg
Injection, atezolizumab, 10 mg
Injection, avelumab, 10 mg
Injection, azacitidine, 1 mg
Vaccine for bladder cancer injection into urinary bladder
Bcg live intravesical 1mg
Injection, bendamustine hcl (treanda), 1 mg
Injection, bendamustine hcl (bendeka), 1 mg
Injection, bendamustine hydrochloride, (belrapzo/bendamustine) 1 mg
Injection, bevacizumab, 10 mg
Injection, bevacizumab-awwb, biosimilar, (mvasi), 10 mg
Injection, bleomycin sulfate, 15 units
Injection, blinatumomab, 1 microgram
Injection, bortezomib (velcade), 0.1 mg
Injection, bortezomib, not otherwise specified, 0.1 mg
Busulfan; oral, 2 mg
Injection, busulfan, 1 mg
Injection, carboplatin, 50 mg

[1] https://www.cms.gov/medicare/prescription-drug-coverage/prescriptiondrugcovcontra/downloads/partsbdcoveragesummarytable_041806.pdf

© 2022 Kyle MA et al. *JAMA Network Open*.

Injection, carfilzomib, 1 mg
Injection, carmustine, 100 mg
Injection, cemiplimab-rwlc 1 mg
Injection, cetuximab, 10 mg
Injection, cladribine, per 1 mg
Injection, cisplatin, powder or solution, 10 mg
Cyclophosphamide; oral, 25 mg
Cyclophosphamide, 100 mg
Injection, cytarabine, 100 mg
Cytarabine P/F
Dacarbazine, 100 mg
Injection, daratumumab, 10 mg
Injection, decitabine, 1 mg
Injection, degarelix, 1 mg
Injection, docetaxel, 1 mg
Injection, doxorubicin hydrochloride, 10 mg
Injection, doxorubicin hydrochloride, liposomal, not otherwise specified, 10 mg
Injection, durvalumab, 10 mg
Injection, elotuzumab, 1 mg
Injection, eribulin mesylate, 0.1 mg
Etoposide; oral, 50 mg
Injection, etoposide, 10 mg
Injection, fludarabine phosphate, 50 mg
Injection, fluorouracil, 500 mg
Injection, fulvestrant, 25 mg (faslodex)
Injection, gemcitabine hydrochloride, 200 mg
Goserelin acetate implant, per 3.6 mg
Injection, inotuzumab ozogamicin, 0.1 mg
Injection, irinotecan, 20 mg
Injection, irinotecan liposome, 1 mg
Injection, ixabepilone, 1 mg
Injection, lanreotide, 1 mg
Injection, leuprolide acetate (for depot suspension), per 3.75 mg
Leuprolide acetate (for depot suspension), 7.5 mg
Eligard
Lupron Depot
Lupaneta Pack
Melphalan; oral, 2 mg
Injection, melphalan hydrochloride, 50 mg
Methotrexate; oral, 2.5 mg (J8610)
Methotrexate (J9250)*
Methotrexate (J9260)*
Methotrexate Sodium
Otrexup (methotrexate)
Rasuvo (methotrexate)
Injection, mitomycin, 5 mg
Injection, mitoxantrone hydrochloride, per 5 mg

© 2022 Kyle MA et al. *JAMA Network Open*.

Injection, mogamulizumab-kpkc 1 mg
Injection, nivolumab, 1 mg
Injection, obinutuzumab, 10 mg
Injection, octreotide, depot form for intramuscular injection, 1 mg
Injection, ofatumumab, 10 mg
Injection, olaratumab, 10 mg
Injection, oxaliplatin, 0.5 mg
Injection, paclitaxel, 1 mg
Injection, paclitaxel protein-bound particles, 1 mg
Injection, panitumumab, 10 mg
Injection, pembrolizumab, 1 mg
Injection, pertuzumab, 1 mg
Injection, pralatrexate, 1 mg
Injection, ramucirumab, 5 mg
Injection, rituximab, 10 mg
Injection, rituximab 10 mg and hyaluronidase
Injection, romidepsin, 1 mg
Injection, siltuximab, 10 mg
Topotecan, oral, 0.25 mg
Injection, topotecan, 0.1 mg
Injection, trabectedin, 0.1 mg
Injection, trastuzumab, 10 mg
Injection, triptorelin pamoate, 3.75 mg
Injection, vinblastine sulfate, 1 mg
Injection, vincristine sulfate liposome, 1 mg
Vincristine sulfate, 1 mg
**Part D primary**
Injection, omacetaxine mepesuccinate, 0.01 mg

© 2022 Kyle MA et al. *JAMA Network Open*.

eReferences

1. *Use of Pharmacy Benefit Managers and Efforts to Manage Drug Expenditures*. United States Government Accountability Office; 2019.

2. Jacobson G, Cicchiello A, Sutton JP, Shah A. *Medicare Advantage vs. Traditional Medicare: How Do Beneficiaries' Characteristics and Experiences Differ?* Commonwealth Fund; 2021. doi:10.26099/yxq0-1w42

3. Anderson KE, Polsky D, Dy S, Sen AP. Prescribing of low- versus high-cost Part B drugs in Medicare Advantage and traditional Medicare. *Health Serv Res*. n/a(n/a). doi:10.1111/1475-6773.13912

4. Schwartz AL, Zlaoui K, Foreman RP, Brennan TA, Newhouse JP. Health Care Utilization and Spending in Medicare Advantage vs Traditional Medicare: A Difference-in-Differences Analysis. *JAMA Health Forum*. 2021;2(12):e214001. doi:10.1001/jamahealthforum.2021.4001

5. Centers for Medicare and Medicaid Services. Oncology Care Model. Accessed April 25, 2022. https://innovation.cms.gov/innovation-models/oncology-care

© 2022 Kyle MA et al. *JAMA Network Open*.
